# Clinical Performance Evaluation of VersaTrek 528 Blood Culture System in a Chinese Tertiary Hospital

**DOI:** 10.3389/fmicb.2018.02027

**Published:** 2018-08-28

**Authors:** Pinli Yue, Menglan Zhou, Timothy Kudinha, Xiuli Xie, Juan Du, Hongmei Song, Lintao Zhang, Xiaojun Ma, Li Weng, Wenzhao Chai, Huadong Zhu, Qiwen Yang, Ying-Chun Xu

**Affiliations:** ^1^Department of Clinical Laboratory, Peking Union Medical College Hospital, Peking Union Medical College, Chinese Academy of Medical Sciences, Beijing, China; ^2^Beijing Key Laboratory for Mechanisms Research and Precision Diagnosis of Invasive Fungal Diseases, Beijing, China; ^3^Department of Clinical Laboratory, Charles Sturt University, Orange, NSW, Australia; ^4^Pathology West, NSW Health Pathology, Orange, NSW, Australia; ^5^Department of Infectious Diseases, Peking Union Medical College Hospital, Peking Union Medical College, Chinese Academy of Medical Sciences, Beijing, China; ^6^Department of Medical Intensive Care Unit, Peking Union Medical College Hospital, Peking Union Medical College, Chinese Academy of Medical Sciences, Beijing, China; ^7^Department of Intensive Care Unit, Peking Union Medical College Hospital, Peking Union Medical College, Chinese Academy of Medical Sciences, Beijing, China; ^8^Department of Emergency Medicine, Peking Union Medical College Hospital, Peking Union Medical College, Chinese Academy of Medical Sciences, Beijing, China

**Keywords:** VersaTrek 528, FX 400, blood culture system, clinical evaluation, simulated blood culture, bloodstream infection

## Abstract

**Background:** The aim of this study was to evaluate the clinical performance of VersaTrek 528 compared to BACTEC FX 400 blood culture (BC) systems.

**Materials and Methods:** Simulated and clinically obtained BCs were used in the study. Confirmed bacterial species (*n* = 78), including 43 Gram-positives, 30 Gram-negatives, and 5 *Candida albicans* strains, were each inoculated into BC bottles. Clinically obtained BCs were subdivided into two groups, A and B. In group A were 72 BC sets (pair: aerobic and anaerobic) in which a set inoculated with 5 ml blood was processed in the VersaTrek BC system, whilst the one inoculated with 10 ml blood was processed in the FX BC system. In group B, 76 BC sets (pairs) corresponding to 152 VersaTrek bottles and 152 FX bottles were inoculated with the same volume (10 ml) of blood, and processed in each system.

**Results:** In the simulated BC study, 90% (63/70) of the VersaTrek aerobic bottles were positive, which was higher than that of FX 400 (59/70, 84%), but was not statistically significant (*P* = 0.423). In contrast, FX 400 anaerobic bottles had a higher positive rate than the other BC system (84 vs. 77%), although it was statistically insignificant (*P* = 0.267). Time to detection of organisms in the two BCs was comparable for both aerobic (*P* = 0.131) and anaerobic bottles (*P* = 0.104). In clinical BCs of group A, FX BC system had slightly higher positive rates for both aerobic (11.1 vs. 9.7%, *P* = 0.312) and anaerobic (8.3 vs. 6.9%, *P* = 0.375) bottles. However, the difference was not statistically significant. In group B, VersaTrek aerobic bottles had a higher positive rate compared to the other BC system (10.5 vs. 5.2%, *P* = 0.063). In terms of positive rates of sub-studies A and B, VersaTrek and FX BC systems were comparable.

**Conclusion:** There was no significant difference between the two BC systems in the detection of bacteria and fungi in simulated BCs. In clinical BCs, the performance of the VersaTrek BC system, with inoculation of 5 or 10 ml patient’s blood, was comparable to the FX system with inoculation of 10 ml patient’s blood.

## Introduction

In spite of the great advances in medical science in the past century, bloodstream infection still remains a growing public health problem worldwide (Goto and Al-Hasan, 2013). Detection of microorganisms in the blood is important to identify and isolate pathogens in clinical laboratories, allowing clinicians to optimize patient’s treatment (Jacobs et al., 2017). Blood culture (BC) is among the most common microbiological tests, and remains the gold standard for detection of bloodstream infections (Burd and Kehl, 2011; Lamy et al., 2016). Over the past few decades, improvements in BC media and the availability of software-assisted automated growth detectors have promoted the recovery of bloodstream pathogens, and decreased the time to detection (TTD) of microbial growth (Fiori et al., 2014). Effective utilization of BC systems can help clinicians initiate appropriate antimicrobial treatment to patients within 24 h of BC positivity.

The VersaTrek 528 is an automated BC microbial detection system produced by ThermoScientific, and is based on detection of pressure changes due to gas consumption (O_2_) and/or production (CO_2_, N_2_, H_2_, etc.) by microorganisms in a BC bottle. It is a more advanced and automated instrument compared to the single detection of CO_2_ production routinely used in several BC systems. Unlike most BC systems, VersaTrek 528 can detect bacterial pathogens that consume O_2_ without CO_2_ production. So it can provide a more credible and reliable result, improve recovery and reduce the TTDs of significant pathogens.

The BD BACTEC FX 400 instrument is an automated system for detecting the presence of microorganisms in clinical samples. This system is based on the utilization of carbohydrate substrates in BC media, and continuous production of CO_2_ by growing microorganisms, which has a positive correlation with fluorescence intensity. It detects the growth of microorganisms through a fluorescence sensor set at the bottom of the BC bottle (Kirn and Weinstein, 2013).

This study represents the first evaluation of the performance of the VersaTrek 528 BC system in detection and TTDs of bacteria and fungi, compared with BACTEC FX 400 BC system, using simulated and clinically obtained BCs.

## Materials and Methods

### Bacteria and Equipment

The study was performed at Peking Union Medical College Hospital (PUMCH), a tertiary hospital in Beijing, China. The study was carried out in accordance with the institute’s guidelines and procedures, including ethics approval by the Human Research Ethics Committee (Ethics Approval No. S-736).

All the clinical isolates and BC specimens used in this study were collected at PUMCH. The 78 bacterial species used in the simulation study were isolated from positive BCs and stored at -80°C until use. Identification of these isolates was performed by matrix-assisted laser desorption/ionization time-of-flight mass spectrometry (MALDI-TOF MS) and 16S-rRNA gene sequencing. The 146 clinical BC specimens were collected from adult patients with suspected bloodstream infections during the period 2016–2017. The bacterial species included in the simulation study represent the majority of bacteria isolated from BCs at PUMCH.

Thermo Fisher Scientific VersaTrek 528 BC system and BD FX 400 BC system were used in the study. The VersaTrek 528 aerobic BC bottles (VT-S) and VersaTrek 528 anaerobic BC bottles (VT-F) were incubated in the VersaTrek 528 BC system. FX 400 aerobic BC bottles (FX-S) and FX 400 anaerobic BC bottles (FX-F) were incubated in the FX 400 BC system.

### Simulated Blood Cultures

Several bacterial isolates (each obtained from a different patient) from previous blood stream infections (*n* = 78) were used in the simulated BC study, including *Staphylococcus aureus* (*S. aureus, n* = 5), *Staphylococcus epidermidis* (*S. epidermidis, n* = 5), *Enterococcus faecalis* (*E. faecalis, n* = 5), *Enterococcus faecium* (*E. faecium, n* = 5), *Streptococcus agalactiae* (*S. agalactiae, n* = 5), *Streptococcus pneumoniae* (*S. pneumoniae, n* = 5), *Streptococcus pyogenes* (*S. pyogenes, n* = 5), *Streptococcus mitis* (*S. mitis, n* = 5), *Escherichia coli* (*E. coli, n* = 5), *Klebsiella pneumoniae* (*K. pneumoniae, n* = 5), *Enterobacter cloacae* (*E. cloacae, n* = 5), *Pseudomonas aeruginosa* (*P. aeruginosa, n* = 5), *Acinetobacter baumannii* (*A. baumannii, n* = 5), *Bacteroides fragilis* (*B. fragilis, n* = 5), *Peptostreptococcus* spp. (*n* = 3), *Candida albicans* (*C. albicans, n* = 5). All the isolates were previously identified by standard MALDI-TOF MS method and 16S-rRNA sequencing.

The 78 clinical bacterial isolates were used in the simulated BC study. Isolates were recovered from frozen stocks and cultured overnight on appropriate agar medium at 37°C and 5% CO_2_. Colonies from agar plates were re-suspended in stroke-physiological saline solution to 0.5 McFarland (bacteria: 1.5 × 10^8^ CFU/ml, Candida: 10^6^ CFU/ml) and diluted to a final concentration of approximately 10 CFU/ml. One milliliter from the last suspension was inoculated in both aerobic and anaerobic BC bottles for each of the two BC systems as described previously (Almuhayawi et al., 2015), i.e., VersaTrek 528 aerobic BC bottle (VT-S), VersaTrek 528 anaerobic BC culture bottle (VT-F), FX 400 aerobic BC bottle (FX-S) and FX 400 anaerobic BC bottle (FX-F) vials. The BC bottles were each inoculated with 5 ml sterile human blood prior to inoculation with previously obtained organisms. Seventy clinical bacteria isolates were inoculated in aerobic BC bottles of each of the two BC systems under study, and 73 isolates were inoculated in both anaerobic BC bottles of the two BC systems (*B. fragilis* and *Peptostreptococcus* spp. isolates were only inoculated in anaerobic BC bottles; *C. albicans* isolates were only inoculated in aerobic BC bottles).

### Clinical Blood Culture Specimens

**FIGURE 1:**
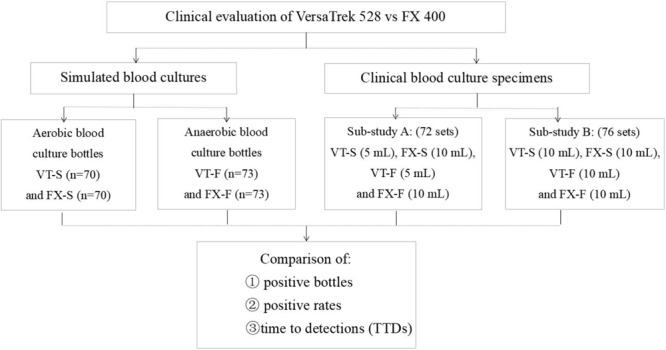
The experimental flow chart and grouping status in the study. *Bacteroides fragilis* and *Peptostreptococcus* spp. isolates were only inoculated in anaerobic BC bottles; *Candida albicans* isolates were only inoculated in aerobic BC bottles. In sub-study A, 5 ml of blood was inoculated into VersaTrek 528 blood culture (BC) bottles (aerobic and anaerobic) and 10 ml of blood was inoculated into FX 400 BC bottles (aerobic and anaerobic). In sub-study B, 10 ml of blood was inoculated into VersaTrek 528 and FX 400 BC bottles (aerobic and anaerobic). VT-S means aerobic bottles of VersaTrek 528 BC system; FX-S means aerobic bottles of FX 400 BC system; VT-F means anaerobic bottles of VersaTrek 528 BC system; FX-F means anaerobic bottles of FX 400 BC system.

Clinical BCs (*n* = 148), collected from adult patients with suspected bacteremia or fungemia, during the period 2016–2017, were included in the study. Skin or access ports were disinfected with alcohol to reduce the contamination rate (Garcia et al., 2015). Blood was collected by peripheral venipuncture rather than by intravenous catheter (Dawson, 2014). The clinical BC study specimens were grouped into two, A and B. In group A were 72 clinical BCs collected from the emergency department and Medical Intensive Care Unit. For these BCs, 15 ml of blood was aseptically collected from the left arm and inoculated into one VersaTrek 528 aerobic BC bottle (VT-S: 5 ml) and one FX 400 aerobic BC bottle (FX-S: 10 ml). A further 15 ml of blood was collected from the right arm and inoculated into one VersaTrek 528 anaerobic BC bottle (VT-F: 5 ml) and one FX 400 anaerobic BC bottle (FX-F: 10 ml). Group B consisted of 76 BCs collected from the Intensive Care Unit and Infectious Disease wards. For these BCs, 20 ml of blood was aseptically collected from the left arm and inoculated into one VersaTrek 528 aerobic BC bottle (VT-S: 10 ml) and one FX 400 aerobic BC bottle (FX-S: 10 ml). Another 20 ml was collected from the right arm and inoculated into one VersaTrek 528 anaerobic BC bottle (VT-F: 10 ml) and one FX 400 anaerobic BC bottle (FX-F: 10 ml). The collected BC bottles were quickly transported to the laboratory and loaded in both BC systems. The experimental flow chart and grouping status are shown in **Figure 1**.

### Conventional Methods

Blood culture bottles inoculated with blood from patients were incubated in the respective BC system until signaling for positivity or for a maximum of 5 days (Altun et al., 2016). BCs flagged positive for microbial growth were Gram stained and the results immediately reported to the patient’s physician. According to the Gram stain results, the positive BCs were sub-cultured onto relevant agar plates, and any growing organisms were identified by MALDI-TOF MS and 16S-rRNA sequencing. A BC that flagged positive, had organisms seen on Gram stain, and grew on subculture, was considered a true positive. BCs that were flagged positive but showed no organism on the Gram smear and no growth on subculture were re-incubated. BCs that were still negative at the end of the 5th day of incubation were also terminally sub-cultured. Those that were persistently negative for subculture were classified as false-positive detections and excluded from analysis (Fiori et al., 2014). Bottles that did not signal positive at the end of 5 days were sub-cultured on agar plates (48 h in at 37°C) for confirmation.

### Definitions and Data Analyses

The positive rates for the two BC system were compared by using the Chi-square test of McNemar. Yates’correction was used when n was less than 40. The TTD was compared by the Wilcoxon matched-pairs signed-rank test. Values of *P* < 0.05 were considered to be statistically significant. Statistical analyses were performed using GraphPad Prism 7.

## Results

### Simulated Blood Cultures

In total, 140 aerobic and 146 anaerobic BC bottles were studied in two different BC systems, using the same clinical bacteria (*n* = 78) isolates (*B. fragilis* and *Peptostreptococcus* spp. isolates were only inoculated in anaerobic BC bottles; *C. albicans* isolates were only inoculated in aerobic BC bottles). During the 5 day incubation period, 90% (63/70) VT-S, 84% (59/70) FX-S, 77% (56/73) VT-F, and 84% (61/73) FX-F BC bottles, signaled positive in the two BC systems (**Supplementary Table S1**). There was no significant difference in the detection of organisms between VT-S and FX-S (90 vs. 84%, *P* = 0.423). Likewise, there was no significant difference in the detection of organisms between VT-F and FX-F (77 vs. 84%, *P* = 0.267). **Table 1** shows the detection of bacteria in both BC systems by Gram stain category, bacterial species, and aerobic status. There was no significant difference in the detection rate of organisms among Gram-positive, Gram-negative and fungal isolates between the two BC systems. VersaTrek 528 aerobic bottles showed a higher positive rate for Gram-positive bacteria (92.5%) compared to the other method (80%), but the difference was not statistically significant (*P* = 0.182). Likewise, FX 400 anaerobic bottles showed a higher positive rate for Gram-negative bacteria (63.3 vs. 70%, *P* = 0.683) with no significant statistical difference.

**Table 1:** Comparison of the detection of organisms in simulated blood culture bottles for two BC systems.

Micoorganism (no)	Aerobic bottle (n)				*P*	Anaerobic bottle (n)				*P*
	VT only^d^	FX only^e^	Both^f^	Neither^g^		VT only	FX only	Both	Neither	
Gram-positive (43)	7	2	30	1	NS^a^	2	5	35	1	NS
*S. mitis* (5)	0	0	5	0	NA^b^	0	0	5	0	NA
*S. pyogenes* (5)	2	0	3	0	NS	0	2	3	0	NS
*S. pneumoniae* (5)	1	1	2	1	NS	1	0	3	1	NS
*S*. *agalactiae* (5)	2	0	3	0	NS	0	0	5	0	NA
*S. epidermidis* (5)	1	0	4	0	NS	0	2	3	0	NS
*S. aureus* (5)	0	0	5	0	NA	0	0	5	0	NA
*Peptostreptococcus* spp. (3)	NT	NT	NT^c^	NT	NA	1	1	1	0	NS
*E. faecium* (5)	1	0	4	0	NS	0	0	5	0	NA
*E. faecalis* (5)	0	1	4	0	NS	0	0	5	0	NA
Gram-negative (30)	2	3	19	1	NS	2	4	17	7	NS
*E. coli* (5)	1	0	4	0	NS	0	2	2	1	NS
*K. pneumoniae* (5)	0	1	4	0	NS	0	0	5	0	NA
*E. cloacae* (5)	0	2	3	0	NS	0	0	5	0	NA
*P. aeruginosa* (5)	1	0	3	1	NS	1	2	0	2	NS
*B. fragilis* (5)	NT	NT	NT	NT	NA	0	0	5	0	NA
*A. baumannii* (5)	0	0	5	0	NA	1	0	0	4	NS
Yeast (5)	0	0	5	0	NA	NT	NT	NT	NT	NA
*C. albicans* (5)	0	0	5	0	NA	NT	NT	NT	NT	NA
Total (78)	9	5	54	2	NS	4	9	52	8	NS

*^*a*^NS, not significant. ^*b*^NA, not applicable. ^*c*^NT, not test. ^*d*^The bottles only signaled positive in VersaTrek 528 blood culture system. ^*e*^The bottles only signaled positive in FX 400 blood culture system. ^*f*^The bottles signaled positive both in VersaTrek 528 and FX 400 blood culture systems. ^*g*^The bottles signaled positive neither in VersaTrek 528 nor FX 400 blood culture systems.*

Three isolates (1 each of *E. coli, P. aeruginosa*, and *Peptostreptococcus* spp.) were only detected in VersaTrek 528 BC system, and no one isolate was only detected in the FX BC system. At the single-species level, there were no significant differences in the detection of organisms between the two BC systems (**Table 1**). Nine bacterial species (*S. aureus, S. epidermidis, E. faecium, S. agalactiae, S. pyogenes, S. mitis, E. coli, A. baumannii, C. albicans*) had a 100% (5/5) detection rate in VT-S. There were 7 bacterial species (*S. aureus, E. faecalis, S. mitis, K. pneumoniae, E. coli, A. baumannii, C. albicans*) with 100% (5/5) detection rate in FX-S. Eight species (*S. aureus, E. faecalis, E. faecium, S. agalactiae, S. mitis, K. pneumoniae, E. coli, B. fragilis*) had a detection rate of 100% (5/5) in VT-F, and 10 species (*S. aureus, S. epidermidis, E. faecalis, E. faecium, S. agalactiae, S. pyogenes, S. mitis, K. pneumoniae, E. coli, B. fragilis*) had a 100% (5/5) detection rate in FX-F. *S. aureus* and *S. viridians* were both detected (100%, 5/5) in all four BC bottle types. The results of Gram-stain and MALDI-TOF MS identifications were concordant with the previously confirmed identity of the isolates inoculated, without any false positives and contaminated BC bottles.

**FIGURE 2:**
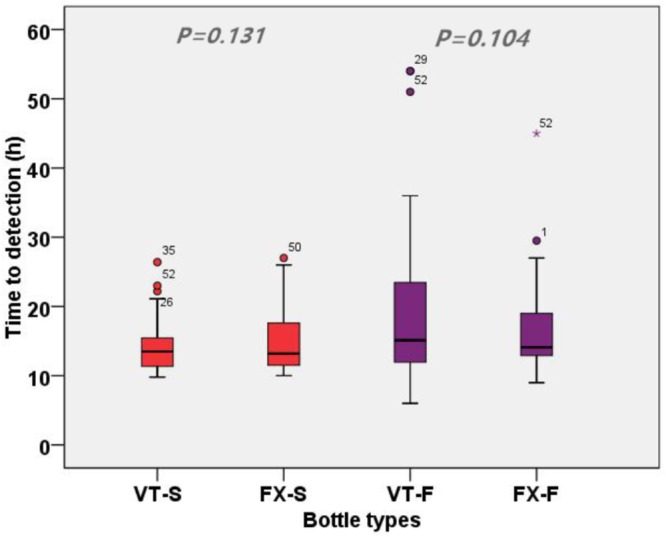
Time to detections (TTDs) of the isolates that grew in two types aerobic or two types anaerobic BC bottles. VT-S means aerobic bottles of VersaTrek 528 BC system; FX-S means aerobic bottles of FX 400 BC system; VT-F means anaerobic bottles of VersaTrek 528 BC system; FX-F means anaerobic bottles of FX 400 BC system.

The performance of the specific BC bottles in terms of TTD is shown in **Figure 2**. The TTDs for the positive VT-S (*n* = 63) and FX-S (*n* = 59) bottles were similar ([median, 13.8; IQR, 11.4–16.0] vs. [median, 13.1; IQR, 11.3–18.0], *P* = 0.8). Also, the TTDs for the positive VT-F (*n* = 56) and FX-F (*n* = 61) bottles were similar ([median, 15.2; IQR, 12.0–23.9] vs. [median, 14.2; IQR, 12.3–19.2]; *P* = 0.310). The median TTD for FX-F bottles was shorter than that of VT-F. The 54 isolates that signaled positive in both VT-S and FX-S bottles, and the 52 isolates that signaled positive in both VT-F and FX-F, were used in TTD comparison analysis (**Figure 2**). There was no significant difference in the TTDs of VT-S and FX-S ([median, 13.8; IQR, 11.4–16.1] vs. [median, 13.7; IQR, 11.5–18.0]; *P* = 0.131, *n* = 54). Likewise, there was no significant difference in the TTDs of VT-F and FX-F bottles ([median, 15.1 IQR, 11.9–23.7] vs. [median, 14.1; IQR, 12.9–19.0]; *P* = 0.104, *n* = 52). This suggests that VT-S has a similar median TTD with FX-S, and FX-F has a shorter median TTD than VT-F (*P* = 0.104). In all, TTDs of the two BC systems were comparable for both aerobic (*P* = 0.131) and anaerobic bottles (*P* = 0.104).

### Clinical Blood Culture Specimens

The organisms detected in the two BC systems are shown in **Table 2**. There were 15 (15/148, 10%) positive bottles in VT-S, 12 (12/148, 8%) in FX-S, 5 (5/148, 3%) in VT-F, and 7 (7/148, 5%) in FX-F. There was no significant difference in the detection of organisms in aerobic bottles (10 vs. 8%, *P* = 0.508) and anaerobic bottles (3 vs. 5%, *P =* 0.625) of the two BC systems. False positive results were recorded in 2 VT-S and 3 VT-F bottles which flagged positive, but the Gram stain yielded nothing and there was no growth in sub-cultures on relevant agar plates. In group A study, the FX BC system detected more isolates in both the aerobic (11.1 vs. 9.7%, *P* = 0.312) and anaerobic (8.3 vs. 6.9%, *P* = 0.375) bottles compared to the other BC system. In sub-study B, VersaTrek 528 aerobic bottles had a higher positive rate than the other system (10.5 vs. 5.2%, *P* = 0.063). There was only one positive anaerobic bottle in FX BC system, and none in VersaTrek 528 BC system (**Table 3**). In terms of positive rates of sub-studies A and B, VersaTrek 528 and FX BC systems were comparable.

**Table 2:** The characteristics of positive BC bottles in the clinical sample study.

Patient No.	Blood set No.	Blood volume (ml)		VT-S bottles		FX-S bottles		TD^d^	VT-F bottles		FX-F bottles		TD
		VT	FX	TTD (h)	ID^c^	TTD (h)	ID	VTS-FXS (h)	TTD (h)	ID	TTD (h)	ID	VTF-FXF (h)
P1	BS1	10	10	12	*A. baumannii*	NEG	NA	NA^b^	NEG	NA	NEG	NA	NA
	BS2	10	10	8	*A. baumannii*	8	*A. baumannii*	0	NEG	NA	NEG	NA	NA
	BS3	10	10	8	*A. baumannii*	9	*A. baumannii*	–1	NEG	NA	NEG	NA	NA
P2	BS4	10	10	10	*P. aeruginosa*	NEG	NA	NA	NEG	NA	NEG	NA	NA
P3	BS5	10	10	6.5	*E. coli*	6	*E. coli*	0.5	NEG	NA	7	*E. coli*	NA
P4	BS6	10	10	43	*S. felis + S. cohnii*	NEG	NA	NA	NEG	NA	NEG	NA	NA
P5	BS7	10	10	26	*S. hominis*	NEG	NA	NA	NEG	NA	NEG	NA	NA
P6	BS8	10	10	48	*B. cepacia*	29	*B. cepacia*	19	NEG	NA	NEG	NA	NA
P7	BS9	5	10	21	*S. hominis*	27	*S. hominis*	–6	NEG	NA	NEG	NA	NA
P8	BS10	5	10	NEG	NA	23	*T. asahii*	NA	NEG	NA	NEG	NA	NA
P9	BS11	5	10	NEG	NA	NEG	NA	NA	NEG	NA	15	*sgc*	NA
P10	BS12	5	10	20	*E. coli*	11	*E. coli*	9	68	*E. coli*	10	*E. coli*	58
	BS13	5	10	14	*E. coli*	NEG	NA	NA	NEG	NA	12	*E. coli*	NA
P11	BS14	5	10	NEG	NA	16	*E. coli*	NA	NEG	NA	NEG	NA	NA
P12	BS15	5	10	8	*K. pneumoniae + E. coli*	8	*K. pneumoniae + E. coli*	0	13	*K. pneumoniae + E. coli*	7	*K. pneumoniae + E. coli*	6
P13	BS16	5	10	24	*K. pneumoniae*	18	*K. pneumoniae*	6	171	*K. pneumoniae*	11	*K. pneumoniae*	160
P14	BS17	5	10	NEG	NA	12	*E. coli*	NA	14	*E. coli*	11	*E. coli*	3
P15	BS18	5	10	9	*A. baumannii*	9	*A. baumannii*	0	34	*A. baumannii+ P. aeruginosa*	NEG	NA	NA
P16	BS19	5	10	46	*C. tuberculostearicum*	NEG	NA	NA	NEG	NA	NEG	NA	NA
P17	BS20	5	10	NEG	NA	NEG	NA	NA	102	NEG (false positive)	NEG	NA	NA
P18	BS21	5	10	NEG	NA	NEG	NA	NA	119	NEG (false positive)	NEG	NA	NA
P19	BS22	5	10	120	NEG (false positive)^a^	NEG	NA	NA	NEG	NA	NEG	NA	NA
P20	BS23	5	10	83	NEG (false positive)	NEG	NA	NA	70	NEG (false positive)	NEG	NA	NA

*^*a*^False positive, there was no microorganism after Gram stain and subculturing onto relevant agar plates specimens from the positive bottles. ^*b*^NA, not applicable. ^*c*^ID means the identifications of microorganisms in signaled positive bottles. ^*d*^TD means time difference which is the TTDs of VersaTrek 528 blood culture bottles minus the TTD of FX 400 blood culture bottles.*

**Table 3:** Microorganisms recovery comparisons of the two blood culture systems in sub-group A and sub-group B.

Bottle types	No.	Aerobic bottles (n)				*P*	Anaerobic bottles (n)				*P*
		VT only	FX only	Both	Neither		VT only	FX only	Both	Neither	
Substudy A^a^	72	2	3	5	62	NS^c^	1	2	4	65	NS
Substudy B^b^	76	4	0	4	68	NS	0	1	0	75	NS
Total	148	6	3	9	130	NS	1	3	4	140	NS

*^*a*^Substudy A: there were 5 ml blood inoculated in VersaTrek 528 BC bottles and 10 ml blood inoculated in FX 400 BC bottles. ^b^Substudy B: there were 10 ml blood inoculated in VersaTrek 528 BC bottles and 10 ml blood inoculated in FX 400 BC bottles. ^*c*^NS, *P* > 0.05, not significant.*

The bacterial isolates recovered from the positive bottles included 8 *A. baumannii*, 2 *P. aeruginosa*, 17 *E. coli*, 2 *Burkholderia cepacia* (*B. cepacia*), 3 *Staphylococcus hominis* (*S. hominis*), 1 *Trichosporon asahii* (*T. asahii*), 8 *K. pneumoniae*, 1 *S. agalactiae*, 1 *Staphylococcus felis* (*S. felis*), 1 *Staphylococcus cohnii* (*S. cohnii*), and 1 *Corynebacterium tuberculostearicum* (*C. tuberculostearicum*). There were 6 positive bottles which grew two different bacterial species, indicating a mixed infection. In sub-study A, 3 positive BCs (including 1 each yielding *T. asahii, S. agalactiae*, and *E. coli*) were only detected in the FX BC system, and no BC was only positive in the VersaTrek 528 BC system. In sub-study B, 5 BCs (each yielding one of the following, *P. aeruginosa, S. hominis, C. tuberculostearicum*, and *S. felis* mixed with *S. cohnii*), were only positive in VersaTrek 528 BC system, and no BC was positive only in the FX BC system (**Table 2**). There were 3 BCs which were only positive in the VT-S system (BS6, BS7 and BS19, corresponding to 1 *S. felis* mix with *S. cohnii*, 1 *C. tuberculostearicum*, and 1 *S. hominis*), which were classified as contaminations.

## Discussion

For decades, BC has been considered the “gold standard” for detecting bacteremia (Almuhayawi et al., 2015). Many laboratories use a set of aerobic and anaerobic bottles per specimen in routine blood culturing (Mirrett et al., 2004; Coorevits and Van den Abeele, 2015). Clinical microbiology laboratory directors and supervisors must frequently face the problem of selecting the optimal BC system for use in their laboratory (Mirrett et al., 2003). The aim of this study was to compare the performance of VersaTrek 528 and FX 400 BC systems.

In the simulated BC study, we analyzed 78 commonly encountered clinical bacterial isolates. After 5 days incubation, VersaTrek 528 aerobic BC bottles had a higher detection rate than FX 400 aerobic BC bottles (*P* = 0.423), and FX 400 anaerobic BC bottles had a higher detection rate than VersaTrek 528 anaerobic BC bottles (*P* = 0.267). However, the differences were not statistically significant. This suggests that despite differences in BC systems, they generally have similar performances in detecting microorganisms. Furthermore, there were no significant differences in the detection of Gram-positive, Gram-negative bacteria and fungi, between the two BC systems. In terms of TTDs for the two BC systems, they were comparable for both aerobic (*P* = 0.131) and anaerobic bottles (*P* = 0.104). Rapid detection of growth in BCs is critically important as it guides the initiation of appropriate antibacterial therapy early. However, the number of isolates studied for certain bacterial species was relatively small to draw definite conclusions, although some trends were observed. Overall, there was no significant difference in the performance of the two BC systems in simulated BC.

In clinical BC specimens, we grouped the specimens into groups A and B in order to analyze the performance of the two BC systems based on the blood volume used. Since the number of microorganisms circulating in the bloodstream may be comparatively small, the size of the blood volume used strongly affects BC sensitivity and TTDs. Indeed, the blood volume inoculated in a BC, is the most important factor influencing the detection of bloodstream microorganisms as bacterial or fungal density in the bloodstream is very low in a majority of patients with bloodstream infections (Lamy et al., 2016). Overall, the possibility of detecting bloodstream infections depends on the concentration and volume of bacteria and fungi in the blood (Arpi et al., 1989). However, getting sufficient volume of blood (10 ml) to fill a set of BC bottles from a single venipuncture may be difficult, especially in the elderly, young, and patients with shock. However, in this study, there was no significant difference between VersaTrek 528 and FX 400 BC systems, in both sub-study A and sub-study B. This suggests that the VersaTrek 528 BC system which used an inoculation volume of 5 or 10 ml blood, performed equally (in the detection of organisms) to the BACTEC FX 400 BC system which used an inoculation volume of 10 ml blood. No significant difference was observed in sub-study A (*P* = 0.375) and sub-study B (*P* = 0.063). These findings suggest that the performance of the VersaTrek system with inoculation of 5 or 10 ml patient’s blood is comparable to FX 400 system with inoculation of 10 ml patient’s blood. Due to limited number of positive BC bottles in the study, we didn’t compare TTDs of these bottles in the clinical sample study group.

Five false positive bottles were detected in the VersaTrek 528 BC system, but none in the FX 400 BC system. High leukocyte counts, over-filled bottles and/or errors in incubation, are major causes of false positive BCs (Reimer et al., 1997). However, there is a scarcity of data on the possible causes of BC false positives. The causes for false positive bottles in the VersaTrek 528 BC system still need to be explored. There were three bottles with contaminants in the VersaTrek 528 BC system despite the progress in skin antisepsis. These included 1each of *S. felis* mixed with *S. cohnii, C. tuberculostearicum* and *S. hominis.* These organisms are known colonizers of the skin and were detected only in one bottle of a BC set.

This study has some limitations. The first is that compared with the *in vitro* environment of a septic patient (who may have used antibiotics), we didn’t add antimicrobial agents in the simulated BC study. Secondly, the number of positive bottles in the clinical BC study group was small, and thus we didn’t compare TTDs of the positive bottles. Finally, due to the small number of fungal isolates during the course of the study, we cannot make conclusions regarding the performance of the two blood BC systems for fungi.

## Conclusion

There was no significant difference between the two BC systems in the detection of organisms. The VersaTrek system with inoculation of 5 or 10 ml patient’s blood, was comparable to the BACTEC FX 400 system with inoculation of 10 ml patient’s blood. Further clinical studies are warranted to investigate the feasibility of inoculating 5 ml patient’s blood instead of 10 ml for VersaTrek 528 BC bottles in clinical practice, and how to reduce the rate of false positive bottles and contaminants.

## Author Contributions

QY and Y-CX conceived and designed the work. MZ, XX, JD, HS, LZ, and QY performed the survey. PY and TK analyzed the data and wrote the manuscript. XM, LW, HZ, and WC collected the clinical samples. All authors read and approved the final manuscript.

## Conflict of Interest Statement

The authors declare that the research was conducted in the absence of any commercial or financial relationships that could be construed as a potential conflict of interest.
