# Supplementary material for: Clinical Performance Evaluation of VersaTrek 528 Blood Culture System in a Chinese Tertiary Hospital
Source: Front Microbiol. 2018 Aug 28;9:2027. doi: 10.3389/fmicb.2018.02027 (PMC6120971; doi:10.3389/fmicb.2018.02027)
Supplement: Supplementary file 1 [file Table_1.DOCX]

Comparison of time to detection of different bacterial isolates in simulated blood cultures between two blood culture systems

| Number | Microorganism | TTD(h) | | |  | TTD(h) | | |
| --- | --- | --- | --- | --- | --- | --- | --- | --- |
|  |  | VT-S^d^ | FX-S^e^ | VT-FX^f^ |  | VT-F^g^ | FX-F^h^ | VT-FX^i^ |
| 1 | *S. aureus* | 15.1 | 12.1 | 3 |  | 15.1 | 29.5 | -14.4 |
| 2 | *S. aureus* | 13.4 | 11.2 | 2.2 |  | 16.1 | 11.4 | 4.8 |
| 3 | *S. aureus* | 15.2 | 12.4 | 2.8 |  | 15.2 | 16 | -0.9 |
| 4 | *S. aureus* | 14.1 | 11 | 3.1 |  | 12.6 | 14.8 | -2.2 |
| 5 | *S. aureus* | 13.2 | 17 | -3.8 |  | 13.2 | 13.4 | -0.2 |
| 6 | *S. epidermidis* | 15.6 | NEG^a^ | NA^c^ |  | NEG | 29.5 | NA |
| 7 | *S. epidermidis* | 16 | 18 | -2 |  | NEG | 19 | NA |
| 8 | *S. epidermidis* | 14 | 18 | -4 |  | 30 | 18 | 12 |
| 9 | *S. epidermidis* | 15 | 18 | -3 |  | 27 | 20 | 7 |
| 10 | *S. epidermidis* | 15 | 18 | -3 |  | 34 | 19 | 15 |
| 11 | *E. faecalis* | NEG | 10.4 | NA |  | 10.6 | 13.2 | -2.7 |
| 12 | *E. faecalis* | 11.2 | 10.6 | 0.6 |  | 10.6 | 13.2 | -2.7 |
| 13 | *E. faecalis* | 13.4 | 10 | 3.4 |  | 10.2 | 12.8 | -2.6 |
| 14 | *E. faecalis* | 14.1 | 11 | 3.1 |  | 12.6 | 16 | -3.4 |
| 15 | *E. faecalis* | 13.6 | 11.5 | 2.1 |  | 15.4 | 11.3 | 4.1 |
| 16 | *E. faecium* | 11.8 | NEG | NA |  | 15 | 13.1 | 1.9 |
| 17 | *E. faecium* | 14 | 15 | -1 |  | 54 | 13 | 41 |
| 18 | *E. faecium* | 13 | 16 | -3 |  | 18 | 14 | 4 |
| 19 | *E. faecium* | 13 | 14 | -1 |  | 25 | 13 | 12 |
| 20 | *E. faecium* | 11 | 12 | -1 |  | 13 | 11 | 2 |
| 21 | *S. agalactiae* | 9.8 | 18.2 | -8.4 |  | 34.8 | 9.5 | 25.3 |
| 22 | *S. agalactiae* | 10 | NEG | NA |  | 12 | 10 | 2 |
| 23 | *S. agalactiae* | 10 | NEG | NA |  | 11 | 10 | 1 |
| 24 | *S. agalactiae* | 10 | 12 | -2 |  | 12 | 11 | 1 |
| 25 | *S. agalactiae* | 10 | 10 | 0 |  | 12 | 9 | 3 |
| 26 | *S. pneumoniae* | 13.4 | 13.1 | 0.3 |  | 13.3 | NEG | NA |
| 27 | *S. pneumoniae* | NEG | 13.5 | NA |  | 14.6 | 15.5 | -0.9 |
| 28 | *S. pneumoniae* | 16.5 | 14.9 | 1.6 |  | 15.1 | 17.5 | -2.4 |
| 29 | *S. pneumoniae* | NEG | NEG | NA |  | NEG | NEG | NA |
| 30 | *S. pneumoniae* | 16.7 | NEG | NA |  | 23.9 | 24.7 | -0.8 |
| 31 | *S. pyogenes* | 13.8 | NEG | NA |  | NEG | 11.1 | NA |
| 32 | *S. pyogenes* | 14 | 13 | 1 |  | NEG | 11 | NA |
| 33 | *S. pyogenes* | 12 | 13 | -1 |  | 36 | 12 | 24 |
| 34 | *S. pyogenes* | 13 | 13 | 0 |  | 21 | 11 | 10 |
| 35 | *S. pyogenes* | 12 | NEG | NA |  | 54 | 13 | 41 |
| 36 | *S. mitis* | 22.2 | 21 | 1.2 |  | 19.1 | 27 | -7.9 |
| 37 | *S. mitis* | 21.1 | 20.8 | 0.3 |  | 19.3 | 24.6 | -5.3 |
| 38 | *S. mitis* | 20 | 21 | -1 |  | 20 | 23 | -3 |
| 39 | *S. mitis* | 12.5 | 14.5 | -2 |  | 15.1 | 14.3 | 0.8 |
| 40 | *S. mitis* | 15.7 | 19.4 | -3.7 |  | 18.7 | 17.3 | 1.4 |
| 41 | *E. coli* | 11.4 | 11.5 | -0.1 |  | NEG | 12.3 | NA |
| 42 | *E. coli* | 30.6 | NEG | NA |  | NEG | NEG | NA |
| 43 | *E. coli* | 11.2 | 11.7 | -0.5 |  | NEG | 12.3 | NA |
| 44 | *E. coli* | 14.1 | 13.3 | 0.8 |  | 11.2 | 13.9 | -2.7 |
| 45 | *E. coli* | 11.3 | 17.2 | -5.9 |  | 11.9 | 9.4 | 2.5 |
| 46 | *K. pneumoniae* | 26.4 | 10.2 | 16.2 |  | 10.3 | 13 | -2.8 |
| 47 | *K. pneumoniae* | NEG | 11.6 | NA |  | 10.4 | 14.2 | -3.9 |
| 48 | *K. pneumoniae* | 14.1 | 11.8 | 2.3 |  | 10.4 | 14.2 | -3.9 |
| 49 | *K. pneumoniae* | 10.6 | 10.6 | 0 |  | 11.1 | 13.8 | -2.7 |
| 50 | *K. pneumoniae* | 10.9 | 16.2 | -5.3 |  | 12.7 | 9.4 | 3.3 |
| 51 | *E. cloacae* | NEG | 11.2 | NA |  | 6 | 13.4 | -7.4 |
| 52 | *E. cloacae* | 11.4 | 11.2 | 0.2 |  | 10.4 | 14.2 | -3.8 |
| 53 | *E. cloacae* | NEG | 10.6 | NA |  | 9.5 | 16.8 | -7.3 |
| 54 | *E. cloacae* | 14 | 11.3 | 2.7 |  | 14.6 | 19.3 | -4.7 |
| 55 | *E. cloacae* | 11.4 | 11.6 | -0.2 |  | 20.5 | 13.4 | 7.1 |
| 56 | *P. aeruginosa* | 17.5 | 15.4 | 2.1 |  | NEG | 33.8 | NA |
| 57 | *P. aeruginosa* | 17.5 | 16 | 1.5 |  | NEG | 23 | NA |
| 58 | *P. aeruginosa* | 17 | 15.4 | 1.6 |  | NEG | NEG | NA |
| 59 | *P. aeruginosa* | NEG | NEG | NA |  | NEG | NEG | NA |
| 60 | *P. aeruginosa* | 14.7 | NEG | NA |  | 23.9 | NEG | NA |
| 61 | *A. baumannii* | 10.4 | 23.3 | -12.9 |  | 23 | NEG | NA |
| 62 | *A. baumannii* | 10 | 11 | -1 |  | NEG | NEG | NA |
| 63 | *A. baumannii* | 10 | 11 | -1 |  | NEG | NEG | NA |
| 64 | *A. baumannii* | 11 | 16 | -5 |  | NEG | NEG | NA |
| 65 | *A. baumannii* | 12 | 13 | -1 |  | NEG | NEG | NA |
| 66 | *B. fragilis* | NT^b^ | NT | NA |  | 27.5 | 21.4 | 6.1 |
| 67 | *B. fragilis* | NT | NT | NA |  | 27 | 20 | 7 |
| 68 | *B. fragilis* | NT | NT | NA |  | 24 | 19 | 5 |
| 69 | *B. fragilis* | NT | NT | NA |  | 23 | 20 | 3 |
| 70 | *B. fragilis* | NT | NT | NA |  | 23 | 19 | 4 |
| 71 | *Peptostreptococcus* spp. | NT | NT | NA |  | 36.7 | NEG | NA |
| 72 | *Peptostreptococcus* spp. | NT | NT | NA |  | 51 | 45 | 6 |
| 73 | *Peptostreptococcus* spp. | NT | NT | NA |  | NEG | 62 | NA |
| 74 | *C. albicans* | 21 | 27 | -6 |  | NT | NT | NA |
| 75 | *C. albicans* | 20 | 25 | -5 |  | NT | NT | NA |
| 76 | *C. albicans* | 23 | 26 | -3 |  | NT | NT | NA |
| 77 | *C. albicans* | 21 | 23 | -2 |  | NT | NT | NA |
| 78 | *C. albicans* | 26 | 27 | -1 |  | NT | NT | NA |

a Bottle was negative at the end of 5-day incubation.

b The isolation was not tested in that blood culture bottle.

c NA, not applicable.

d VT-S means aerobic bottles of VersaTrek 528 blood culture system.

e FX-S means aerobic bottles of FX 400 blood culture system.

f VT-FX means the TTD of VersaTrek 528aerobic blood culture bottle minus the TTD of FX 400 aerobic blood culture bottle.

g VT-F means anaerobic bottles of VersaTrek 528 blood culture system.

h FX-F means anaerobic bottles of FX 400 blood culture system.

i VT-FX means the TTD of VersaTrek 528 anaerobic blood culture bottle minus the TTD of FX 400 anaerobic blood culture bottle.
